# A New Sampling Method for Spleen Stiffness Measurement Based on Quantitative Acoustic Radiation Force Impulse Elastography for Noninvasive Assessment of Esophageal Varices in Newly Diagnosed HCV-Related Cirrhosis

**DOI:** 10.1155/2014/365982

**Published:** 2014-03-04

**Authors:** Leonardo Rizzo, Massimo Attanasio, Marilia Rita Pinzone, Massimiliano Berretta, Michele Malaguarnera, Aldo Morra, Luca L'Abbate, Luca Balestreri, Giuseppe Nunnari, Bruno Cacopardo

**Affiliations:** ^1^Ultrasuoni Diagnostic Medical Center, 95100 Catania, Italy; ^2^Department of Mathematics and Statistics “S. Vianelli”, University of Palermo, viale delle Scienze, 90128 Palermo, Italy; ^3^Department of Clinical and Molecular Biomedicine, Division of Infectious Diseases, University of Catania, Via Palermo 636, 95125 Catania, Italy; ^4^Department of Medical Oncology, National Cancer Institute IRCCS, Via Franco Gallini 2, 33081 Aviano, Italy; ^5^Euro-Mediterranean Institute of Science and Technology (IEMEST), Via Emerico Amari 123, 90139 Palermo, Italy; ^6^Research Center “The Great Senescence”, University of Catania, 95125 Catania, Italy; ^7^Radiological Diagnostic Center, “Euganea Medica”, 35100 Padova, Italy; ^8^Division of Radiology, National Cancer Institute IRCCS, Via Franco Gallini 2, 33081 Aviano, Italy

## Abstract

In our study, we evaluated the feasibility of a new sampling method for splenic stiffness (SS) measurement by Quantitative Acoustic Radiation Force Impulse Elastography (Virtual Touch Tissue Quantification (VTTQ)).We measured SS in 54 patients with HCV-related cirrhosis of whom 28 with esophageal varices (EV), 27 with Chronic Hepatitis C (CHC) F1–F3, and 63 healthy controls. VTTQ-SS was significantly higher among cirrhotic patients with EV (3.37 m/s) in comparison with controls (2.19 m/s, *P* < 0.001), CHC patients (2.37 m/s, *P* < 0.001), and cirrhotic patients without EV (2.7 m/s, *P* < 0.001). Moreover, VTTQ-SS was significantly higher among cirrhotic patients without EV in comparison with both controls (*P* < 0.001) and CHC patients (*P* < 0.01). The optimal VTTQ-SS cut-off value for predicting EV was 3.1 m/s (AUROC = 0.96, sensitivity 96.4%, specificity 88.5%, positive predictive value 90%, negative predictive value 96%, positive likelihood ratio 8.36, and negative likelihood ratio 0.04). In conclusion, VTTQ-SS is a promising noninvasive and reliable diagnostic tool to screen cirrhotic patients for EV and reduce the need for upper gastrointestinal endoscopy. By using our cut-off value of 3.1 m/s, we would avoid endoscopy in around 45% of cirrhotic subjects, with significant time and cost savings.

## 1. Introduction

Chronic hepatitis C virus (HCV) infection represents a worldwide health concern, with 170 million chronically infected subjects, whose risk of developing cirrhosis within 20 years is estimated to be around 10% to 20% [1, 2]. Portal hypertension (PH) is associated with the most severe complications of cirrhosis, such as ascites, hepatic encephalopathy, and bleeding from esophageal varices (EV). Gastroesophageal varices are present in approximately 50% of patients with liver cirrhosis [3–5]. The most accurate method to evaluate PH is the measurement of the hepatic vein pressure gradient (HVPG). It has been demonstrated that a HVPG value higher than 10 mmHg predicts the presence of EV, while a value higher than 12 mmHg is predictive for variceal bleeding [6]. However, the evaluation of HVPG is an invasive procedure, which is limited to highly specialized centers and experienced operators.

As variceal bleeding is a life-threatening condition, current guidelines recommend routine upper gastrointestinal endoscopy to be performed at the time of diagnosis in all patients with known cirrhosis for EV screening and grading. The endoscopic procedure should allow the identification of high-risk subjects, requiring prophylactic treatment with nonselective beta-blockers. Unfortunately, a number of reasons may limit the universal spread of endoscopic screening in cirrhotic patients. Firstly, universal endoscopic screening is expensive, time consuming, and invasive [7]. Secondly, since the point prevalence of medium/large varices (those requiring a prompt identification) is approximately 15%–25%, the majority of subjects undergoing endoscopic screening either do not have EV or do not require any prophylactic therapy. Furthermore, since only 40% of Child-Pugh class A cirrhotic have EV in comparison with 85% of Child-Pugh class C patients, it seems mandatory to promptly identify the variceal risk in the lower Child-Pugh class cirrhotic population. Thus, many efforts have been made to develop noninvasive surrogate predictive methods for a prompt identification of patients at high risk of EV carriage. Some biochemical markers (aspartate aminotransferase (AST) to platelets ratio index) or mixed indexes (platelets count to spleen diameter ratio) have been shown to partially correlate with the presence of EV [8, 9]. As for imaging techniques, it has been suggested that Magnetic Resonance Imaging and Computed Tomography scan may obviate the need or frequency of endoscopic screening among cirrhotic patients [10, 11]. The ultrasound-based Quantitative Acoustic Radiation Force Impulse Elastography (Virtual Touch Tissue Quantification (VTTQ)) has recently been shown to accurately estimate liver fibrosis by measuring liver stiffness (LS) and could be used for monitoring disease progression or predicting development of liver-related complications; the degree of liver fibrosis is a predictive factor for hepatocellular carcinoma (HCC) development also [12]. Furthermore, it has been suggested that measurement of spleen stiffness (SS) by VTTQ (VTTQ-SS) may predict the presence of EV [13–15]. The spleen of cirrhotic subjects is characterized not only by passive congestion, due to PH, but also by increased fibrosis, angiogenesis, and hyperactivation of the splenic lymphoid compartment [16, 17]. These changes, which are responsible for splenomegaly, may increase SS and may be quantified by transient elastography [18] or VTTQ itself [13–15]. Nevertheless, no data are available on the actual spatial distribution of splenic viscoelasticity in cirrhosis nor standardized models have been proposed for spleen sampling by VTTQ.

The aim of the present study was

- to compare the values of SS of cirrhotic subjects (with and without EV), measured by VTTQ, with those found among healthy controls and patients with HCV-related chronic hepatitis (fibrosis stages F1–F3),
- to evaluate the reliability of VTTQ-SS as a surrogate predictor of EV in a cohort of subjects with recently diagnosed HCV-related cirrhosis.

## 2. Patients and Methods

### 2.1. Cirrhotic Patients

Between January 2009 and January 2011, 73 consecutive patients presenting at the Outpatient Infectious Diseases Unit of the Garibaldi Nesima Hospital in Catania were newly diagnosed as affected with HCV-related liver cirrhosis and considered for enrolment in the present study. In this stage, the diagnosis was based only on a clinical, virological, and ultrasound evaluation.

Subsequently, throughout a 6-month clinical followup, 19 patients were excluded from the study, 17 because of development of severe cirrhosis-related complications or comorbidities (7 patients had hepatocellular carcinoma, 4 severe sepsis, 3 spontaneous bacterial peritonitis, 2 colonic cancer, and 1 lung cancer) and 2 because of refusal to enter the study.

Thus, 54 patients were finally enrolled, 15 with a diagnosis of histologically confirmed Child-Pugh class A cirrhosis and 39 with a clinical diagnosis of Child-Pugh class B cirrhosis. None of the patients had a concomitant HBV and/or HIV coinfection, none had either autoimmune or metabolic disease. Alcohol abuse was excluded by questionnaire. Patients were not under treatment with beta-blockers, diuretics, antibiotics, interferon, or ribavirin by the time of study onset. Moreover, no patient had evidence of cardiac or renal insufficiency. All patients signed a written informed consent prior to study inclusion, in accordance with the Declaration of Helsinki. Patients were asked to hide their clinical features to the ultrasound operator.

Each patient underwent a consecutive 2-day protocol. On the first day, a complete clinical and biochemical evaluation was performed. Then, patients were examined with VTTQ by a single well-experienced operator (LR), blinded to biochemical and clinical data, at the private Outpatient Clinic “Ultrasuoni” in Catania. On the second day, all patients underwent upper gastrointestinal endoscopy. All endoscopic evaluations were performed by a single operator (blinded to biochemical and echographic results) at the Garibaldi Nesima Hospital in Catania.

### 2.2. Chronic Hepatitis C Subjects

We enrolled in the study 27 subjects with a virological and histological diagnosis of chronic hepatitis C (CHC), matched for gender and age distribution with the cirrhotic group. Patients with HBV or HIV coinfection, alcohol abuse, and those under antiviral treatment were excluded from the study. Written informed consent was obtained from each subject at enrolment. Biochemical tests and VTTQ were performed on the same day.

### 2.3. Healthy Controls

63 healthy adult volunteers, matched to cases on age, gender, and ethnic group, were selected as controls among the members of the staff of our institution. They all had normal liver function tests, negative HBsAg, HCV Antibodies, HIV Antibodies, and normal abdominal ultrasonography. All subjects gave their written informed consent prior to study enrolment.

### 2.4. Methods

#### 2.4.1. Quantitative Acoustic Radiation Force Impulse Elastography (VTTQ)

**Figure 1:**
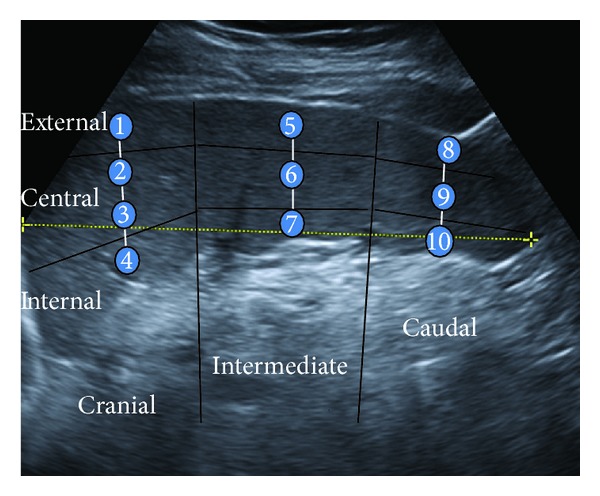
Sites for spleen stiffness measurement by Quantitative Acoustic Radiation Force Impulse Elastography (VTTQ). Cranial section: 1: external, 2 and 3: central, and 4: internal; intermediate section: 5: external, 6: central, and 7: internal; caudal section: 8: external, 9: central, and 10: internal.

B-mode standard ultrasonography scanning and quantitative ARFI elastography (VTTQ) were performed using a Siemens Acuson S2000 (Siemens AG, Erlangen, Germany) with a 4Cl transducer, as described in detail elsewhere [12]. SS was measured with VTTQ only by intercostal approach. The examined patient, fasting for at least 12 hours, was lying in right lateral decubitus. The transducer was perpendicular to the longitudinal axis of the spleen, the line of the VTTQ ROI aligned with the transducer axis. The VTTQ ROI, measuring 10 mm in depth and 5 mm in width, was placed in 10 different sites from the upper to the inferior splenic pole. In order to cover uniformly the spleen, we conducted a stratified random sampling. The stratification variables were (Figure 1)

1. the section, as the spleen was subdivided into three sections, subdiaphragmatic, intermediate, and caudal, and measurements were taken along the corresponding thickness diameter,
2. the part of each section (external, central, and internal).

**Figure 2:**
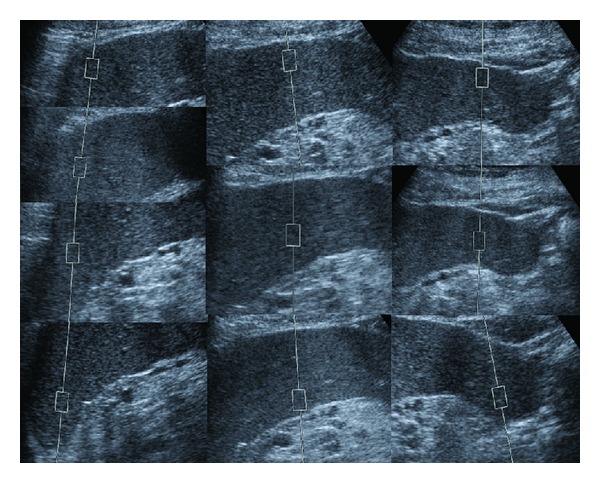
A summary of spleen sampling sites by Quantitative Acoustic Radiation Force Impulse Elastography (VTTQ).

The subdiaphragmatic section provided 4 measurements: 1 medial, 2 central, 1 lateral. The other two sections provided 3 measurements: 1 medial, 1 central, and 1 lateral. This difference was due to the fact that the subdiaphragmatic section was larger. We chose the best intercostal space to maximize the surface area of each section on the frontal plane.  Figure 2 summarizes our sampling method. We then calculated the median value of the 10 measurements.

#### 2.4.2. Interobserver Agreement

Since the procedure could rely on some degree of subjective interpretation by the investigator, we conducted a prior double blind experiment on 16 randomly selected patients among those further enrolled in the study. Briefly, patients were examined for SS by VTTQ as described above by two independent operators within the same day: one operator (LR) was a well-experienced echographiste with a long-term (>4 years) training in ARFI technique, and the other operator (MP) was a young physician who received a prior 4-hour training in ARFI procedure.

#### 2.4.3. Intraobserver Agreement

Twenty-one randomly selected patients among those who entered the study were summoned again to the Outpatient “Ultrasuoni” Clinic four weeks after the first VTTQ-SS examination and submitted to a new VTTQ-SS evaluation by the same experienced operator (LR). The operator, who was still unaware of clinical and endoscopic data, could not access to the previous ARFI results.

#### 2.4.4. Upper Gastrointestinal Endoscopy

Upper gastrointestinal endoscopywas performed using a flexible video gastroscope (Video Pentax Gastro).

EV were classified as follows.

- Grade 1: small straight varices;
- Grade 2: enlarged tortuous varices, occupying less than one third of the lumen;
- Grade 3: large, coil-shaped varices, occupying more than one third of the lumen.

EV were also classified on the basis of presence or absence of red wheals.

### 2.5. Statistical Analysis

Statistical analysis was performed using statistical computing software R and Graphpad Prism 4. Continuous variables are expressed as median (interquartile range, IQR) and compared by nonparametric Kruskal-Wallis test. Categorical variables are presented as number of cases (percentage) and were compared by the *χ* ^2^ test or Fisher's exact test, when appropriate. Correlations between different parameters were analyzed by Spearman correlation coefficients. A logistic regression model was used to assess the impact of SS, spleen diameter and platelet count on the presence of EV.

The best cut-off value to predict the presence of EV was determined by Kolmogorov-Smirnov index, that is, a natural generalization to continuous test of Youden index for binary test [19]. Receiver operating characteristic (ROC) curves were generated to assess the diagnostic performance of VTTQ-SS for the detection of EV. The ROC-curve represents the sensitivity (Se) plotted against 1-specificity (Sp) for all possible cut-off values. The most commonly used index of accuracy is the area under the ROC curve (AUROC), with values close to 1 indicating higher diagnostic accuracy. Finally, we calculated the likelihood ratio (LR) and we graphically represented our data with a Fagan nomogram, which is a graphical tool for estimating how much the result of a diagnostic test changes the probability that a patient has a disease.

Pearson's correlation coefficient was used to evaluate intraobserver agreement; Bland-Altman method was used to assess the agreement between VTTQ-SS measurements obtained by two different observers on the same patients [20].

## 3. Results

### 3.1. Overall Characteristics of Study Population

Table 1 shows the demographic, clinical, echographic, and endoscopic characteristics of the 54 cirrhotic patients enrolled in the present study.

Following upper endoscopy evaluation, 48.2% of cirrhotic patients showed no EV, 20.4% had grade 1 EV, 20.4% had grade 2, and 11% had grade 3. Red wheals were observed in 7.1% of patients with EV. The characteristics of the group of cirrhotic patients with EV and those without EV are shown in Table 2. Platelet count, bilirubin levels, and spleen diameter on sonography significantly differed between the two groups (*P* < 0.05).

As for CHC patients, HCV genotype was 1a/b in 88% of patients, 2 in 3.7%, and 3 in 7.4%. 29.6% of them had F1 fibrosis, 40.8% had F2 fibrosis, and 29.6% had F3 fibrosis. Mean HCV-RNA plasma level was 3.77 ± 1.16  ∗  10^5^ IU/mL.

### 3.2. Comparison of SS by VTTQ in the Subject Groups

**Figure 3:**
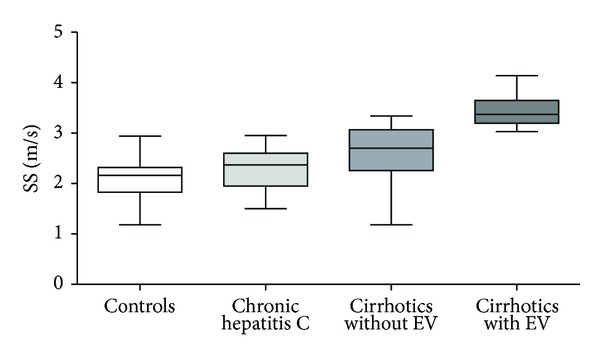
Distribution of spleen stiffness median values measured by Quantitative Acoustic Radiation Force Impulse Elastography (VTTQ) among healthy controls, CHC patients and cirrhotic patients with or without esophageal varices (EV). Splenic stiffness (SS) was significantly higher among cirrhotic patients with EV (3.37 m/s) in comparison with controls (2.19 m/s, *P* < 0.001), CHC patients (2.37 m/s, *P* < 0.001), and cirrhotic patients without EV (2.7 m/s, *P* < 0.001). Moreover, SS was significantly higher among cirrhotic patients without EV in comparison with both controls (*P* < 0.001) and CHC patients (*P* < 0.01).

VTTQ-SS values were higher in cirrhotic patients with EV, when compared with cirrhotic subjects without EV, CHC patients, and controls, as shown in Figure 3. In fact, the median values of spleen stiffness were 2.19 (IQR 1.83–2.31) m/s for controls, 2.37 (IQR 1.96–2.58) m/s for CHC patients, 2.7 (IQR 2.31–3.03) m/s for cirrhotic patients without EV, and 3.37 (IQR 3.21–3.62) m/s for those with EV (*P* < 0.001 versus controls, CHC patients, and cirrhotic subjects without EV). Moreover, VTTQ-SS was significantly higher among cirrhotic patients without EV in comparison with both controls (*P* < 0.001) and CHC patients (*P* < 0.01).

### 3.3. Distribution of SS Median Values

**Figure 4:**
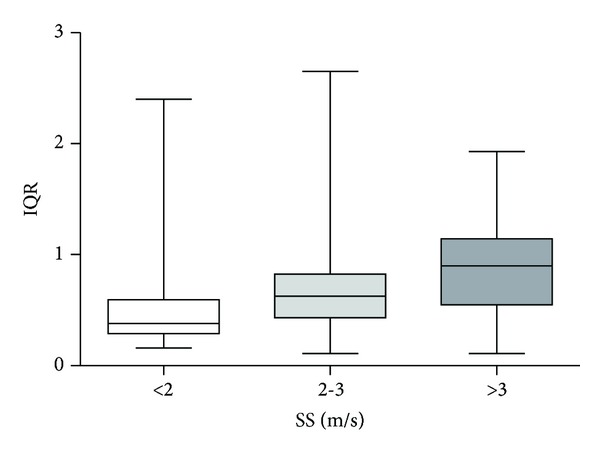
Evaluation of interquartile range (IQR) variability according to spleen stiffness (SS) median values. IQR was significantly higher among subjects with SS median values >3 m/s in comparison with those having SS <2 m/s (*P* < 0.001) or between 2 and 3 m/s (*P* < 0.05). Analogously, IQR was higher among subjects with SS median values between 2 and 3 m/s than those with SS median values <2 m/s (*P* < 0.01).

To evaluate the variability associated with SS measurements, we divided patients into 3 subgroups according to SS median values (SS <2 m/s, 2-3 m/s, >3 m/s) and we found that the IQR was significantly higher among subjects with SS median values >3 m/s in comparison with those having SS <2 m/s (*P* < 0.001) or between 2 and 3 m/s (*P* < 0.05). Analogously, IQR was higher among subjects with SS median values between 2 and 3 m/s than those with SS median values <2 m/s (*P* < 0.01) (Figure 4).

### 3.4. SS Measurement for Predicting EV

**Figure 5:**
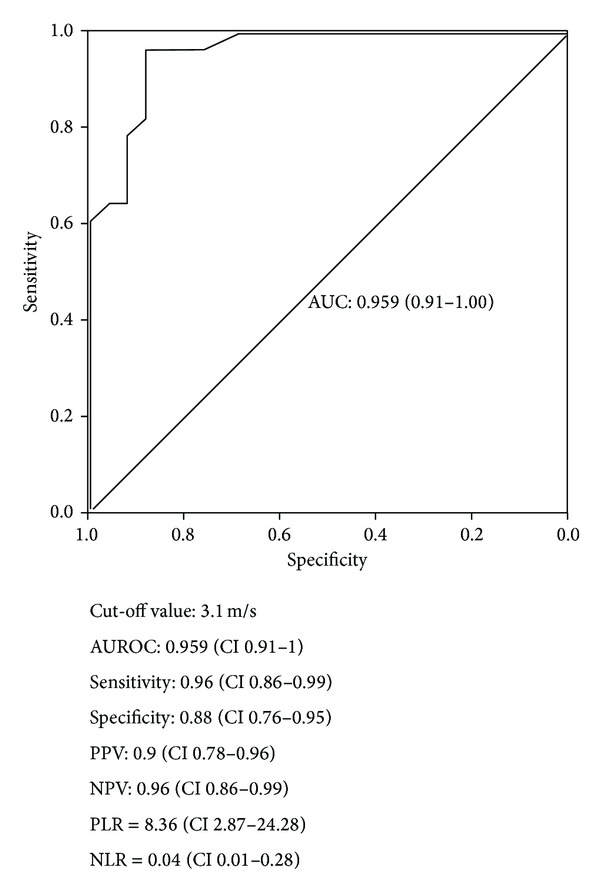
Receiver operating characteristic (ROC) curve of spleen stiffness measured by Quantitative Acoustic Radiation Force Impulse Elastography (VTTQ) for the prediction of esophageal varices. AUROC: area under the ROC curve; CI: confidence interval; NLR: negative likelihood ratio; NPV: negative predictive value; PLR: positive likelihood ratio; PPV: positive predictive value.

The best VTTQ-SS cut-off value to predict the presence of EV, as determined by Kolmogorov-Smirnov index, was 3.1 m/s. The diagnostic accuracy (AUROC) for the prediction of EV was 0.959 (95% confidence interval (CI) 0.91–1), Se 96.4%, Sp 88.5%, positive predictive value (PPV) 90%, negative predictive value (NPV) 96%, positive likelihood ratio (PLR) 8.36, and negative likelihood ratio (NLR) 0.04 (Figure 5).

**Figure 6:**
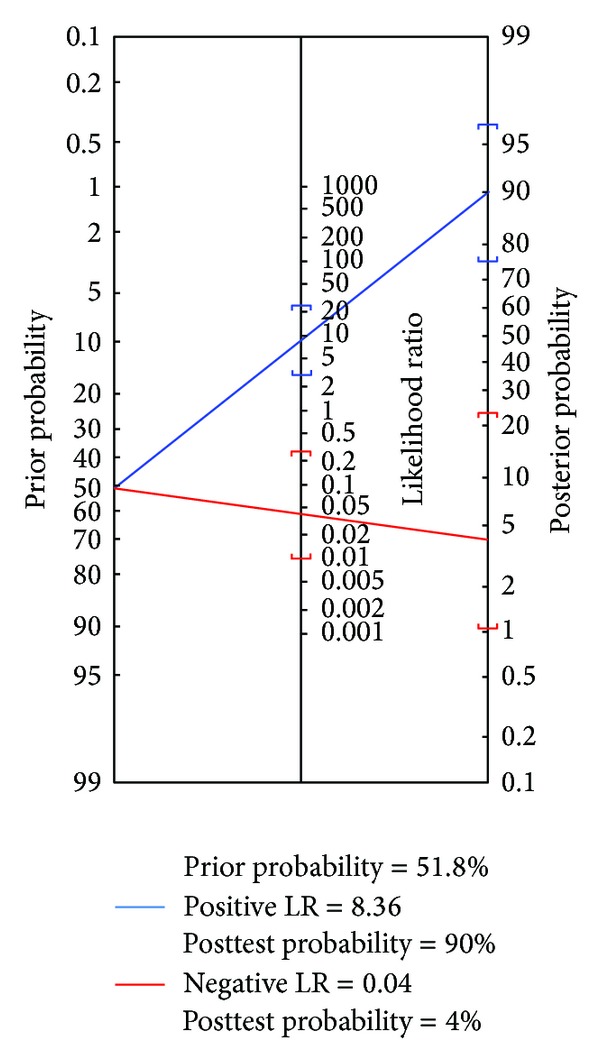
Fagan's Nomogram for esophageal varices (EV). Using the pretest probability for EV of 51.8% (the overall prevalence in our cohort), the posterior probability of having EV was 90%. LR: likelihood ratio; Prior Prob.: pretest (prior) probability; Posterior Prob.: posterior probability.

Figure 6 shows nomographic depiction to estimate posttest probability of having EV from pretest probability and likelihood ratio. Using the pretest probability of 51.8% (the overall prevalence of EV in our cohort), the posttest probability was 90%. With a negative likelihood ratio of 0.04, the posttest probability diminished to 4%.

### 3.5. Relationship between SS, Platelet Count, Spleen Diameter, and EV

In our cohort of cirrhotic patients, SS measured by VTTQ showed a significant negative correlation with platelet count (*r* = −0.328,  *P* = 0.015) but not with spleen diameter (*r* = 0.245,  *P* = 0.073). Furthermore, we found that only SS, but not spleen diameter and platelet count, was associated with the presence of EV (*P* = 0.005) when using a logistic regression model.

### 3.6. Intra- and Interobserver Agreement

**Figure 7:**
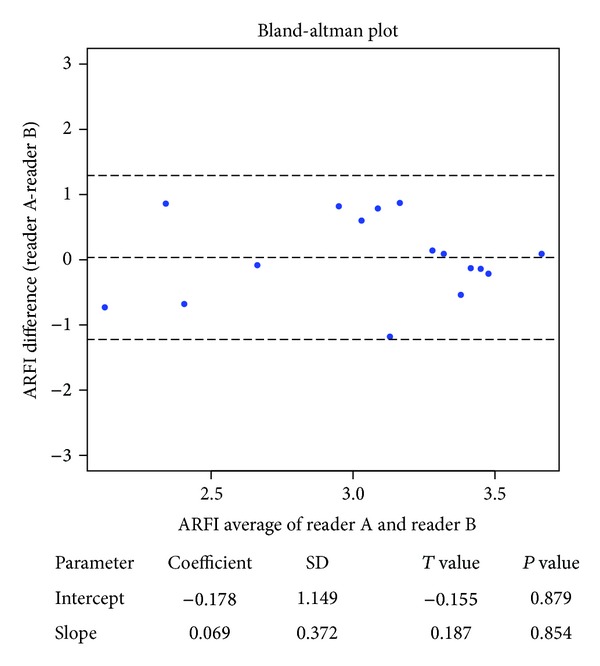
Agreement between Quantitative Acoustic Radiation Force Impulse Elastography (VTTQ) data obtained by two different observers by Bland-Altman method.

By Bland-Altman method, there was no significant difference between VTTQ-SS values of the 16 patients obtained from two different sonographers (Figure 7). Median VTTQ-SS value was 3.3 (IQR 2.7–3.65) m/s for the expert operator and 3.12 (IQR 2.48–3.56) m/s for the novice operator.

Similarly, VTTQ-SS, as evaluated on 21 patients by the same operator in two different time points, was not significantly different (2.97 (IQR 2.52–3.23) m/s for the first measurement versus 2.66 (IQR 2.45–3.33) m/s for the second one). Pearson's correlation coefficient was 0.933 (0.84–0.97).

## 4. Discussion

PH is a common consequence of chronic liver diseases, leading to the formation of esophageal and gastric varices and other severe complications, such as portosystemic encephalopathy and sepsis [3, 4]. HPVG is considered the gold standard for the evaluation of PH and the best surrogate indicator of prognosis in cirrhotic patients [5]. Unfortunately, it is an invasive and expensive procedure, which is not routinely available in clinical practice. According to current guidelines [6], upper gastrointestinal endoscopy is recommended for the detection of EV and the assessment of the bleeding risk. However, considering the invasiveness and costs of both types of investigations, there is a pressing need for new noninvasive surrogate markers. In the present study, VTTQ-SS was characterized by a high diagnostic accuracy for the prediction of EV in cirrhotic patients, independent of related parameters, such as spleen diameter and platelet count. In particular, splenic VTTQ showed an excellent sensitivity (96%) and specificity (88%) and a 96% negative predictive value. Of importance, by applying the cut-off value of 3.1 m/s to the entire population of cirrhotic patients, we would be able to avoid endoscopy in around 45% of them. Our study suggests that VTTQ-SS is able to identify among patients with cirrhosis those who could delay the onset of endoscopic followup, as having a low probability of bearing EV. In fact, in our experience a cut-off value <3.1 m/s was able to accurately rule out the presence of EV, supporting the possibility to reserve upper gastrointestinal endoscopy only to patients with SS >3.1 m/s.

So far, only few reports have assessed the possibility to use ARFI elastography of the spleen as a noninvasive diagnostic tool for the presence of EV [13–15, 21, 22]. In keeping with our observations, Ye et al. have described a significant linear correlation between SS and variceal grade in a cohort of patients with chronic hepatitis B, being 3.16 m/s the optimal cut-off established by the authors for predicting the presence of EV (Se 84.1%, Sp 81%) [21]. In a recently published work of Takuma et al. [15], a cut-off value of 3.18 m/s was able to identify cirrhotic patients with EV with a 98.4% negative predictive value, Se 98.5%, and Sp 60.1%; a cut-off of 3.3 m/s was used to rule out the presence of high-risk varices. Analogously, Bota et al. found SS and LS to be significantly higher in a cohort of newly diagnosed patients with grade 2-3 EV, in comparison with those bearing grade 0-1 EV. Their cut-off for predicting grade 2-3 EV was >2.55 m/s (AUROC 0.578, Se 96.7%, and Sp 47.6%) and the authors elaborated a new score including LS, SS, and the presence of ascites to predict the presence of significant EV (AUROC 0.721, 69.6% accuracy), which was much better than LS and SS alone [13]. These results are in contrast with a previous study of the same group, where the authors failed to detect any significant differences in the mean SS values between cirrhotic patients with and without EV [22].

In comparison with previous reports, our study showed a higher diagnostic performance of VTTQ in predicting EV. A possible explanation is the systematic and extensive sampling of the spleen, which may have been able to adequately represent the heterogeneity of SS distribution. Bota et al. [13] took their measurements 1-2 cm under the capsule, which may not adequately represent the whole splenic parenchyma. Analogously, Ye et al. [21] measured SS in the middle portion of the spleen and 1-2 cm below the splenic capsule, even though their cut-off for predicting EV was quite close to ours (3.16 m/s).

In the study of Takuma et al. [15], SS was measured 1 cm below the spleen capsule; again, although their cut-off (3.18 m/s) did not differ significantly from ours, it should be noticed that their sampling method was different from that proposed in the present study, as they took only five valid measurements for each patient, excluding from further analysis any SS value with an IQR to median value ratio greater than 30% or a success rate less than 60%. The authors did not report the number of excluded measurements. Moreover, this approach did not systematically evaluate the spatial distribution of splenic viscoelasticity and introduces a subjective criterion in the choice of valid measurements. On the contrary, we extensively measured SS throughout the splenic parenchyma in order to assess the variability of SS and obtain a representative sampling of the spleen. In addition, our method demonstrated an excellent reproducibility as assessed by data on inter- and intraobserver agreement. In fact, no significant differences in VTTQ-SS values were found between an expert operator and a novice one, thus demonstrating that VTTQ-SS is easy to perform and does not require a specific training.

Our study has some limitations. Firstly, it was carried out on a relatively small number of subjects. Secondly, only few patients had large EV, so we were not able to identify a cut-off value for large varices. Nevertheless, our preliminary data seem to suggest that VTTQ would work much better and more safely in the selection of cirrhotic patients for endoscopic screening rather than to identify high-risk large-sized varices. Finally, in this preliminary study, we have intentionally omitted to evaluate LS, but it would be worthy for further research to evaluate both LS and SS to validate the combined algorithm suggested by Bota et al. [13] and establish the accuracy of both LS and SS to predict the development of HCC in patients with chronic hepatitis [23–26]. Our findings need further enforcement either by the measurement of HPVG or by an extensive internal and external validation, including patients with cirrhosis of different etiology.

In conclusion, our study suggests that VTTQ-SS could represent an easy-to-perform, noninvasive, reproducible diagnostic tool which can be performed altogether with the routine echographic exam in cirrhotic patients in order to limit and/or delay the indications of endoscopic screening for EV among patients with HCV-related cirrhosis. Further longitudinal studies are needed to confirm our data.

## Figures and Tables

**Table 1:** Demographic, clinical, echographic, and endoscopic characteristics of the 54 patients with HCV-related cirrhosis.

Variables	*N* = 54
Age (years)**	72 (63–79)
Sex, male/female*	25 (46.3)/29 (53.7)
HCV RNA ∗ 10^5^ (IU/mL)^●^	4.92 ± 2.11
HCV genotype 1/2/3/4*	38 (70.4)/4 (7.4)/12 (22.2)/0 (0)
Child-Pugh class A/B*	15 (27.7)/39 (72.3)
Total bilirubin (mg/dL)**	1.3 (0.7–3.1)
AST (IU/L)**	57 (22–111)
ALT (IU/L)**	66 (31–138)
Albumin (g/dL)**	3.8 (2.5–4.1)
Gamma globulins (g/dL)**	2.9 (2.1–3.3)
Platelet count ∗ 10^3^/*μ*L**	111 (34–212)
International normalized ratio (INR)**	1.29 (1–1.98)
Echographic spleen diameter (cm)**	13.9 (9.9–17.8)
Esophageal varices (EV), yes/no*	28 (51.8)/26 (48.2)
EV	
Grade 1*	11 (39.3)
Grade 2*	11 (39.3)
Grade 3*	6 (21.4)
Red wheals, yes/no*	2 (7.1)/26 (92.9)

*Data presented as *N* (%) **Data presented as median (IQR)  ^●^Data presented as mean ± standard deviation.

AST: aspartate aminotransferase; ALT: alanine aminotransferase; IQR: interquartile range.

**Table 2:** Characteristics of cirrhotic patients with esophageal varices (EV) compared to cirrhotic patients without EV by Kruskal-Wallis test.

	Cirrhotics with esophageal varices (*N* = 28)	Cirrhotics without esophageal varices (*N* = 26)
Age (years)**	76 (63–81)	68 (62–75)
Sex, male/female*	13 (46.4)/15 (53.6)	12 (46.1)/14 (53.9)
HCV RNA ∗ 10^5^ (IU/mL)^●^	4.66 ± 2.37	5.11 ± 1.91
HCV genotype 1/2/3/4*	18 (64.3)/2 (7.1)/8 (28.6)/0 (0)	20 (77)/2 (7.7)/4 (15.3)/0 (0)
Child-Pugh class A/B*	8 (28.5)/20 (71.5)	7 (27)/19 (73)
Total bilirubin (mg/dL)**	1.9 (1–3.1)***	1.1 (0.7–2.8)***
AST (IU/L)**	55 (27–111)	58 (21–105)
ALT (IU/L)**	63 (39–136)	69 (30–129)
Albumin (g/dL)**	3.4 (2.1–3.6)	3.9 (2.7–4.1)
Gamma globulins (g/dL)**	3.0 (2.4–3.3)	2.8 (2.1–3.1)
Platelet count ∗ 10^3^/*μ*L**	96 (34–206)***	124 (65–212)***
International normalized ratio (INR)**	1.11 (1–1.59)	1.31 (1.23–1.97)
Echographic spleen diameter (cm)**	14.1 (11.6–17.8)***	11.8 (9.1–12.7)***

*Data presented as *N* (%) **Data presented as median (IQR)  ^●^Data presented as mean ± standard deviation ***P < 0.05.

AST: aspartate aminotransferase; ALT: alanine aminotransferase; IQR: interquartile range.
